# Supplementary material for: Magnetization switching in ferromagnets by adsorbed chiral molecules without current or external magnetic field
Source: Nat Commun. 2017 Feb 23;8:14567. doi: 10.1038/ncomms14567 (PMC5331337; doi:10.1038/ncomms14567)
Supplement: Supplementary Information — Supplementary Figures [file ncomms14567-s1.pdf]

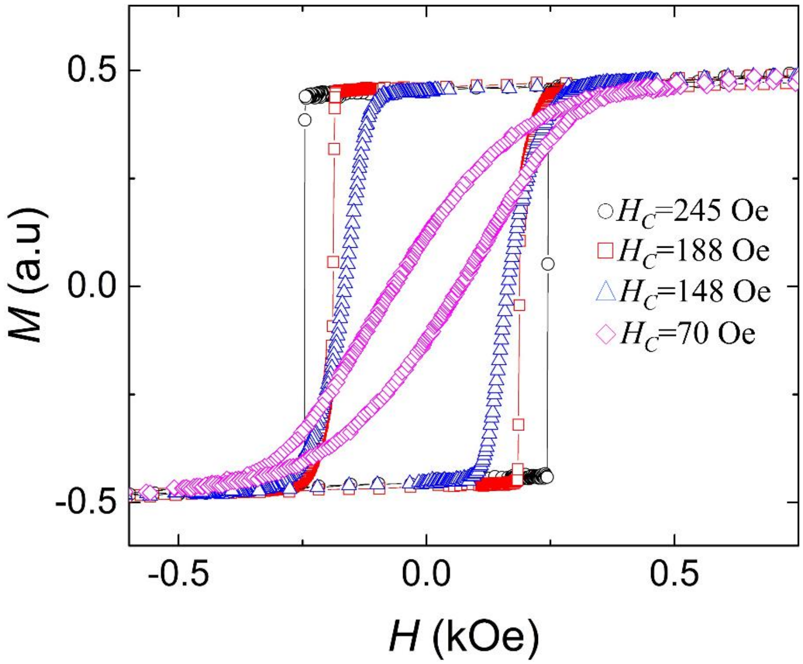

Supplementary Figure 1 | Polar magneto-optic Kerr effect hysteresis loops. Different Co thicknesses correspond to different coercive fields H_C. Maximal H_C=245 Oe is attributed to Co thickness of 1.5 nm, H_C=188 Oe to 1.9 nm, H_C=148 Oe to 2.1 nm and H_C=70 Oe is attributed to Co thickness of 2.2 nm.

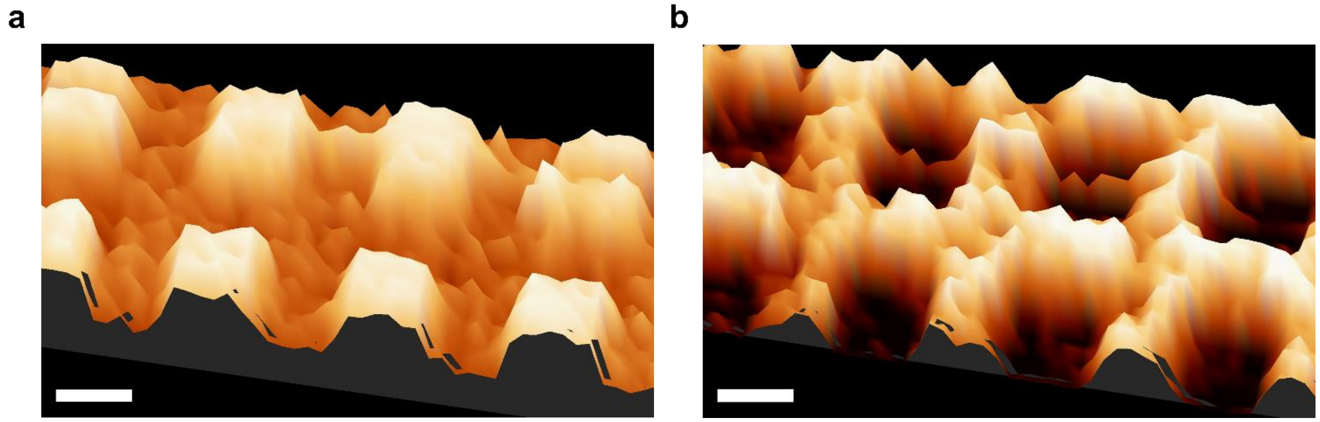

Supplementary Figure 2 | Drop cast adsorption. AFM topography image of 1×1 μm^2 areas of drop-cast AHPA-D molecules (**a**) and their corresponding MFM magnetic phase image (**b**). 4 µl of the 1 mM molecules in ethanol solution were drop-cast on top of the device and later dried in an inert environment overnight. Scale bar is set to 1 µm.

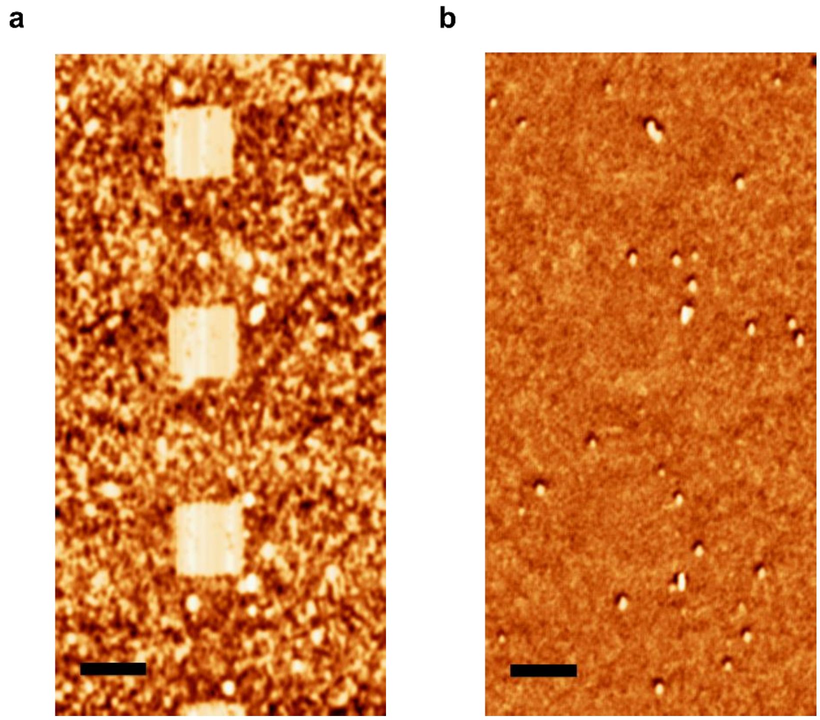

Supplementary Figure 3 | Large coercive field of Co. MFM topography image of 1×1 μm^2 selective adsorption areas of AHPA-L (**a**) and the corresponding magnetic phase image (**b**), displaying the same area as seen in the topography images. The coercive field required for out-of-plane magnetization reorientation is larger than 3000 Oe and therefore MIPAC is barely seen. White spots appearing in both images are PMMA residues with a height above MFM tip scanning elevation height, and therefore represent false magnetic readings. Scale bar is set to 1 μm.

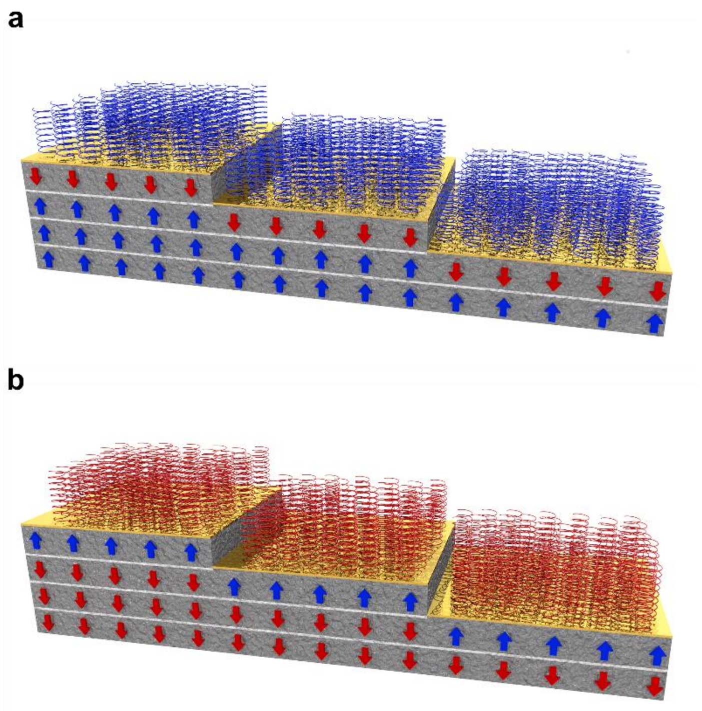

Supplementary Figure 4 | Suggested concept for future application. The fact that MIPAC affects a very thin layer can be utilized for the specific magnetization of one thin layer in a multilayer structure. Selective etching and adsorbing of local AHPA-L (**a**) or AHPA-D (**b**) chiral molecules can be used to create 3D matrices where only the top ferromagnetic layer's magnetization is reoriented whereas the bottom layers' anisotropic magnetization remains unchanged. These matrices can be moved by domain wall motion.
